# AIR POLLUTION: The Oxidative Punch of Wildfires

**DOI:** 10.1289/ehp.117-a58

**Published:** 2009-02

**Authors:** Carol Potera

Thick smoke routinely cloaks Southern California hills, highways, and neighborhoods during this region’s autumn wildfire season, with drought contributing to worse fires than usual in the past two years. Wildfire emissions generate larger aerosols than those produced by vehicles—up to 0.4 micrometers (μm) in diameter compared with less than 0.15 μm—according to a new study led by Constantinos Sioutas, a professor of civil and environmental engineering at the University of Southern California, Los Angeles. In findings published online 6 January 2009 ahead of print in *Environmental Science & Technology*, the larger particles emitted by fires activated a standard assay of oxidative stress even more potently than traffic pollutants. The performance of different types of particulate matter in oxidative stress tests may provide clues as to how these pollutants cause damage such as proinflammatory or cytotoxic effects.

Sioutas and colleagues took advantage of wildfires burning in Southern California in October 2007 to investigate their impact on urban air quality relative to traffic pollutants, with a special emphasis on particulate matter small than 2.5 μm in diameter (PM_2.5_). The researchers collected air samples on the University of Southern California campus, within 150 meters of a busy freeway near downtown Los Angeles. A series of wildfires had started on 20 October 2007, burning more than 500,000 acres of land and stretching from Santa Barbara County to the Mexican border. When air samples were collected on October 24, 25, and 27, fires were raging within 20 miles of the collection site. Measurements were taken from 4:30 p.m. until 10:30 a.m. the following day to capture pollution from evening and morning rush hour traffic as well as from the fires themselves. The fires in Los Angeles County were under control by October 30; post-fire samples collected on November 1 and 14 represented mostly traffic emissions.

Compared with post-fire samples, samples collected on the three fire days in October contained a higher number of particles larger than 0.1 μm in diameter. Both carbon monoxide and nitrogen monoxide levels increased by threefold during fire days, while levels of other air pollutants—ozone, nitrogen dioxide, most polycyclic aromatic hydrocarbons, iron, copper, chromium, zinc, nickel, and vanadium—remained the same. In addition, during the fire period there was an approximate doubling in several indicators for biomass burning: levoglucosan, water-soluble organic carbon, and various minerals (magnesium, phosphorus, potassium, and manganese).

Various chemical components of PM_2.5_ fuel oxidative stress and are toxic to human cells. Working in collaboration with James Schauer of the University of Wisconsin–Madison and Flemming Cassee of the National Institute of Health and the Environment of the Netherlands, the Sioutas team measured cellular toxicity with two standard tests of oxidative stress, the dithiothreitol (DTT) assay and the macrophage reactive oxygen species (ROS) assay. Particles collected during October showed nearly 5 times more DTT activity than those collected in November. In contrast, the macrophage ROS assay, a known indirect measure for combustion pollutants from vehicles that is more responsive to transition metals, peaked in November.

“In this study we demonstrated that a substantial portion of the oxidative potential of air pollution during fires can be attributed to the wildfires,” says Sioutas. He attributes the difference to the increased water-soluble organic compounds that are abundant in wood smoke and show more activity in the DTT assay. According to Sioutas, the elevated levels of water-soluble organic carbon also mean wood smoke particles are more readily absorbed by the body than is PM from traffic sources.

The DTT and macrophage ROS assays “appear to respond to different components of PM_2.5_,” notes Ted Russell, a professor of environmental engineering at the Georgia Institute of Technology in Atlanta. This observation suggests that environmental scientists need to further characterize the two redox assays. A detailed analysis of how these assays respond to specific PM_2.5_ components obtained from different environmental samples at different times “will lay a foundation for understanding the characteristics of ambient PM_2.5_ that have the greatest health impacts,” says Russell.

Sioutas says that the larger particles from wildfires can persist longer in the atmosphere and infiltrate buildings about twice as effectively as traffic pollutants. He recommends that people living near wildfires try to remain inside, shut their doors and windows, and run an air conditioner with a good filter to recirculate the indoor air. “If you don’t have an air conditioner, go to an indoor mall or other public place that recirculates indoor air,” he says. Other practical suggestions are included in the State of California guidelines for wildfire smoke at http://www.arb.ca.gov/smp/progdev/pubeduc/wfgv8.pdf.

## Figures and Tables

**Figure:**
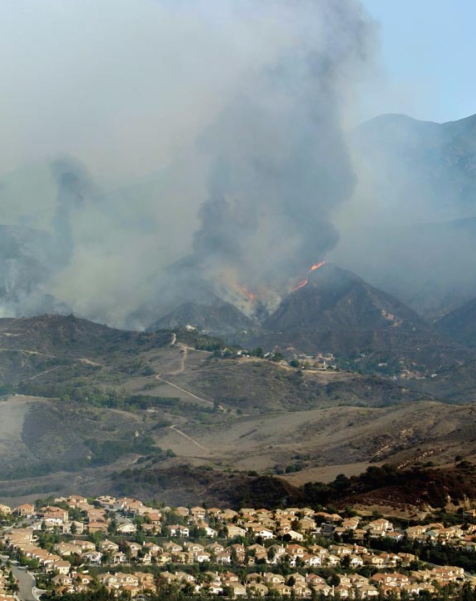
Wildfires burn near a neighborhood in Orange County, California, 24 October 2007.

